# Prevention strategies for hereditary gynaecological cancer in Lynch syndrome

**DOI:** 10.1007/s10689-026-00548-1

**Published:** 2026-03-24

**Authors:** Kevin J. J. Kwinten, Jean-Ellen Johnson, Anne M. van Altena, Nicoline Hoogerbrugge, Emma J. Davidson, Joanne A. de Hullu

**Affiliations:** 1 Department of Obstetrics and Gynecology, Radboud University Medical Center, Nijmegen, The Netherlands; 2 Department of Obstetrics and Gynaecology, St Mary’s Hospital, Manchester University NHS Foundation Trust, Manchester Academic Health Science Centre, Manchester, UK; 3 Department of Human Genetics, Radboud University Medical Center, Nijmegen, The Netherlands; 4 Gynaecological Oncology Research Group, Division of Cancer Sciences, University of Manchester, Faculty of Biology, Medicine and Health, Manchester, UK; 5 Centre for Reproductive Health, Institute for Regeneration and Repair, University of Edinburgh, Edinburgh, UK; 6 CRUK Scotland Centre, Institute of Genetics and Cancer, University of Edinburgh, Edinburgh, UK

**Keywords:** Lynch syndrome, Endometrial cancer, Ovarian cancer, Prevention of cancer, Early detection of cancer

## Abstract

Lynch syndrome is a hereditary cancer predisposition condition associated with an elevated lifetime risk of colorectal, endometrial, ovarian, and several other malignancies. This review provides an updated overview of evidence-based prevention strategies for gynaecological cancers in patients with Lynch syndrome. Risk-reducing hysterectomy with bilateral salpingo-oophorectomy is the most effective intervention for lowering cancer incidence and mortality, but is associated with surgical morbidity and requires careful consideration of reproductive plans and the adverse consequences of premature menopause. Gynaecological surveillance using transvaginal ultrasound and endometrial biopsy is widely implemented as an alternative; however, available evidence is heterogeneous and indicates no benefit in reducing mortality. Novel approaches—such as biomarker-based detection using DNA methylation analysis of cervicovaginal samples, liquid biopsy techniques, and microbiome profiling—offer promising, non-invasive alternatives but require prospective validation in Lynch-specific populations. Chemoprevention with hormonal agents and aspirin may reduce cancer risk, while vaccine-based prevention strategies are under active investigation. Adoption of a healthy lifestyle is recommended for overall health, although its impact on gynaecological cancer risk in Lynch syndrome remains uncertain. Future research should prioritise prospective trials to establish optimal cancer prevention protocols, validate novel biomarkers and preventive cancer vaccine strategies, and evaluate the long-term effectiveness, acceptability, and cost-effectiveness of combined preventive approaches to improve outcomes in this high hereditary-risk population.

## Introduction

Lynch syndrome is an autosomal dominantly inherited cancer predisposition syndrome caused by germline pathogenic variants (gPV) affecting one of the DNA mismatch repair genes—*MLH1*, *MSH2 (*or *EPCAM* deletions*)*, *MSH6*, or *PMS2* [1]. Women with Lynch syndrome have substantially increased lifetime risks of colorectal, endometrial, and ovarian cancer, along with moderately elevated risks of several less common malignancies, including gastric, duodenal, pancreatic, and urinary tract cancers [2].

The incidence of gynaecological cancer varies considerably by mismatch repair gene. By age 75, the cumulative incidences of endometrial and ovarian cancer are estimated at 37% and 8% for individuals with an *MLH1* gPV, 44% and 13% for an *MSH2* gPV, 46% and 6% for an *MSH6* gPV, and 21% and 3% for a *PMS2* gPV, respectively [3].

Prevention strategies aim to reduce cancer risk in patients with Lynch syndrome. Regular colorectal surveillance with colonoscopy facilitates the detection and removal of premalignant lesions, thereby lowering colorectal cancer incidence and mortality [4]. In clinical practice, the prevention of gynaecological cancers in patients with Lynch syndrome has traditionally relied on risk-reducing surgery (RRS) or on gynaecological surveillance when surgery is not yet indicated or not desired [5–7]. Novel approaches, including biomarker-based detection methods, pharmacologic risk-reduction strategies such as hormonal agents or aspirin, and prophylactic vaccination against cancer, are creating new opportunities to advance gynaecological cancer prevention in this high hereditary-risk population.

In this review, we aim to provide an updated overview of evidence-based prevention strategies for endometrial and ovarian cancer in Lynch syndrome.

## Gynaecological surveillance

Although the majority of endometrial cancer cases are diagnosed at an early stage and have a favourable prognosis, with a reported 10-year overall survival rate of 80% [8], this figure still reflects substantial long-term mortality. Treatment generally consists of hysterectomy with bilateral salpingo-oophorectomy with or without pelvic lymph node assessment and is associated with low complication rates [9]. However, in patients with Lynch syndrome who are often diagnosed at a younger age, the consequences of a cancer diagnosis extend beyond treatment-related morbidity. These include psychological distress, reduced quality of life, loss of healthy life years and broader familial, societal and economic implications. Therefore, effective strategies for early detection and prevention are crucial to reduce both mortality and overall burden of disease in this high-risk population.

Schmeler et al. demonstrated that risk-reducing hysterectomy with bilateral salpingo-oophorectomy is the most effective strategy for preventing endometrial cancer [10]. Nonetheless, surgery may not be feasible or carries significant risks for some women due to marked co-morbidities, high anaesthetic risk or technical challenges related to previous abdominal or pelvic procedures. In addition, many women choose to defer or decline RRS in order to preserve their fertility, avoid surgical complications and prevent premature menopause [11]. For these women, gynaecological surveillance may be offered as part of an individualised management approach, either for women who do not undergo RRS or as a temporary measure while awaiting RRS. The goal of surveillance is to identify premalignant lesions and early-stage cancer in asymptomatic women, thus preserving opportunities for fertility-sparing management, reducing treatment-related morbidity, preserve healthy life years and improving survivorship.

A comparison of international guidelines highlights a lack of consensus and substantial variation in whether gynaecological surveillance is recommended, the modalities used, the age at initiation and the recommended interval between assessments (Table 1). This reflects the limited evidence supporting the effectiveness of surveillance in improving cancer detection and survival. A survey of 41 gynaecological oncologists and 298 patients with Lynch syndrome found a substantial variation in gynaecological surveillance services and RRS practices in the United Kingdom [12]. Whilst some NHS providers offer surveillance, others do not, which may be explained by the absence of robust evidence-based guidelines on the management of women with Lynch syndrome and variability in service provision nationally.

**Table 1:** Summary of international guideline recommendations on gynaecological surveillance in Lynch syndrome

Guidelines	ESMO (13)	MICG (5)	NCCN (14)	ESGO-ESTRO-ESP (15)	Dutch national guideline (6)	EviQ (16)	UKCGG (17)
Position on surveillance	May be considered	Not recommended	May be considered	Should be considered	Recommended	Not recommended	Not recommended outside research
Age (years)	30–35		30–35	From 30–35	40–60 (*PMS2* 50–60)		
Surveillance modality	Gynae exam+/-TVS+/- Bx		Bx+/-TVS	TVS+/-Bx	Gynae exam + TVS+Bx		
Interval	Annual		Annual or biennial	Annual or biennial	Annual		
Risk-reducing surgery	RRHBSO after completing childbearing or postmenopause	RRHBSO 35–40 years (*MLH1*, *MSH2*, *MSH6*); insufficient evidence for *PMS2*	RRHBSO ≥ 40 (*MLH1*, *MSH2*); RRHBS ≥ 40 and BO ≥ 50 (*MSH6*); RRHBSO ≥ 50 (*PMS2*)	RRHBSO < 40 (*MLH1*, *MSH2*, *MSH6*); at menopause (*PMS2)*	RRHBSO 40–45 (*MLH1*, *MSH2*, *MSH6*) consider RRHBS postmenopausal (*PMS2*)	RRHBSO ≥ 40 (*MLH1*, *MSH2*, *MSH6*); ≥50 (*PMS2*)	RRHBSO after childbearing ≤ 40 (*MLH1*, *MSH2*, *MSH6*); not routinely recommended premenopause (*PMS2*)

BO, bilateral oophorectomy; Bx, endometrial biopsy; EviQ, EviQ Cancer Treatments Online; ESGO, European Society of Gynaecological Oncology; ESMO, European Society for Medical Oncology; ESP, European Society of Pathology; ESTRO, European Society for Radiotherapy and Oncology; MICG, Manchester International Consensus Group; NCCN, National Comprehensive Cancer Network; RRH, risk-reducing hysterectomy; RRHBS, risk-reducing hysterectomy with bilateral salpingectomy; RRHBSO, risk-reducing hysterectomy with bilateral salpingo-oophorectomy; TVS, transvaginal ultrasound; UKCGG, UK Cancer Genetics Group

Lim et al. conducted a systematic review of 21 studies, of which 18 studies evaluated endometrial cancer surveillance and 14 evaluated ovarian cancer surveillance. The review included both confirmed Lynch syndrome and relatives from Lynch syndrome families undergoing gynaecological surveillance using various modalities, including transvaginal ultrasound, hysteroscopy and endometrial biopsy [18]. The overall incidence of endometrial cancer and ovarian cancer during the surveillance period was 3.9% and 1.3% respectively. Among patients with Lynch syndrome who underwent endometrial cancer surveillance, 64.1% were detected through surveillance, 16.7% were diagnosed in symptomatic women, and 19.2% were not identified through surveillance. The sensitivity of transvaginal ultrasound for endometrial cancer detection was low at 34.4%, and the number needed to screen ranged from 35 to 973 (median 170). However, the study was limited by heterogeneous study populations (combining confirmed patients with Lynch syndrome with those identified on family history alone), few cancer cases, predominantly retrospective studies, and a lack of control groups. The authors concluded that there remains limited evidence to support routine gynaecological surveillance in Lynch syndrome.

**Table 2:** Key studies on gynaecological surveillance in patients with Lynch syndrome. Early surveillance studies predated universal tumour testing and gene-specific risk stratification and often included highly selected families

Study	Design	Age (years)	Surveillance Modality	Comparator	Interval	LS status	Symptomatic	PL	Cancer at screening	FIGO stage	Interval cancer/MC	FIGO stage of interval cancer/MC
**Key earlier surveillance studies (2002–2016)**												
Dove-Edwin (2002), United Kingdom, *N* = 222 (19)	RC	23–68	TA or TVS	No	Annual or Biennial	Mixed^$^	NR	0	0	NA	EC 2	2(I)
Renkonen-Sinisalo (2006), Finland, *N* = 175 (20)	PC	36–66	Varied - TVS+/-TAS+/- Bx+/-CA125	No surveillance. Symptomatic LS with EC. *N* = 83 67 (I), 2 (II), 11 (III), 2 (IV) 1 (NR)	2–3 years	Known LS	0	4	EC 11 (8 by Bx)	9(I), 1(II), 1(III)	EC 2 OC 4	EC 2(II) OC 3(I), 1(III)
Rijcken (2003), Netherlands, *N* = 41 (21)	RC	27–60	TVS+CA125+/-Bx	No	Annual	Mixed^$^	1	3	0	NA	EC 1	1(I)
Lecuru (2008), France, *N* = 57 (22)	PC	Mean age 42	TVS+CA125 + Bx + OPH	No	Annual	Mixed^$^	2	0	EC 2	NR	0	NA
Gerritzen (2009), Netherlands, *N* = 100 (23)	PC	23–72	TVS+/- Bx^1^+/-CA125	No	Annual	Mixed^$^	NR	4	EC 3^2^ OC 2	EC 2(I), 1(III) OC 1(I), 1(III)	0	NA
Jarvinen (2009), Finland, *N* = 103 (24)	PC	18–72	TVS+Bx	No	2–3 years	Known LS	2	0	EC 18 OC 3	EC 13(I), 2(II), 2(III) OC 2(I), 1(II)	EC 6	EC 2(I) OC 2(I), 1(III)
Lecuru (2010), France, *N* = 58 (25)	PC	Mean age 42.5	TVS+/-OPH+/-Bx	No	Annual	Mixed^$^	NR	2	EC 2	NR	0	NA
Guillen-Ponce (2011), Spain, *N* = 91 (26)	RC	NR	TVS+/-Bx	No	Annual	Known LS	NR	NR	EC 3	NR	EC 1^3^	EC 1(I)
Manchanda (2012), United Kingdom, *N* = 41 (27)	PC	Median age 42.9	TVS + OPH+Bx	No	Annual	Mixed^$^	2	1	EC 3	3 (I)	EC 1	EC 1(I)
Stuckless (2013), Netherlands, *N* = 54 (28)	RC	Median age 36	TVS or Bx or CA125	No surveillance *N* = 120 EC 44 OC 16	Annual or Biennial	*MSH2* only	NR	0	EC 5 OC 1	EC 4(I), 1(III) OC 1(II)	EC 4 OC 5	EC 3(I), 1NR OC (I), 2(II), 2NR
Helder-Woolderink (2013), Netherlands, *N* = 75 (29), Period I (TVS+CA125)^7^, Period II (TVS+CA125 + Bx)^8^	RC	26–61	TVS+CA125	Period II Age 23–67 PL 2	Annual	Mixed^$^	NR	4	EC 1	1(I)	0	NA
Ketabi (2014), Denmark, *N* = 871 (30)	RC	19–78	TVS+/-Bx	No	Variable	Mixed^$^	10	3	EC 7 OC 1	EC 5(I), 1(IV), 1NK. OC 1(II)	EC 6 OC 3 AEH 2	EC 3(I), 2(II), 1(III) OC 2(I), 1(III)
Nebgen (2014), USA, *N* = 55 (31)	RC	26–74	TVS+Bx^5^	No	Annual or Biennial	Mixed^$^	0	2	EC 1	1(I)	0	NA
Tzortzatos (2015), Sweden, *N* = 45 (32)	RC	24–84	Various	RRS cohort *N* = 41 EC 3(I) PL 2	1–2 years	*MSH2* only	0	2	EC 3 OC 2	EC 1(I), 2(II) OC 2(I)	EC 4^4^	3(I), 1(II)
Cornou (2016), France, *N* = 177 (33)	PC	Median age 51	TVS+Bx+OPH	No	Annual	Known LS	NR	NR	EC 5 OC 1^6^	NR	0	NA
**Key contemporary surveillance studies (2020–2025)**												
Dueñas (2020), Spain, *N* = 531 (34)	RC	28–80	TVS	RRS cohort *N* = 66 EC 6, 3 pTis, 1(I), 1(II), 1(III)	Annual	Known LS	NR	NR	EC 123 OC 36	EC 57(I), 8(II), 8(III), 4(IV), 46(NR) OC 16(I), 2(II), 7(III), 11(NR)	NR	NA
Eikenboom (2021)*, Netherlands, *N* = 164 (35)	RC	22–75	TVS+/-CA125+/-Bx	RRS cohort Median age 51 *N* = 53 EC 2(I)	Annual	Known LS	5	8	EC 6 OC 1	EC 6(I) OC 1(IV)	0	NA
Eikenboom (2025)*, Netherlands, *N* = 1046 (surveillance *N* = 506, no surveillance *N* = 540) (36)	RC	Median age 56	TVS+/-Bx	No surveillance Median age 65 EC 14, 6(IA) 7(IB or higher) 2(NR) PL 4	Follow up via pathology registry. Surveillance defined as ≥ 2 visits	Known LS	NR	28	EC 37	20(IA), 12(IB or higher), 5(NR)	NR	NR

AEH, atypical endometrial hyperplasia; Bx, endometrial biopsy; EC, endometrial cancer; LS, Lynch syndrome; MC, missed cancers; OC, ovarian cancer; OPH, out-patient hysteroscopy; PC, prospective cohort study; PL, premalignant lesions; RC, retrospective cohort study; RRS, risk-reducing surgery; S, surveillance; TA, transabdominal ultrasound; TVS, transvaginal ultrasound; NA, not applicable; NK, unknown; NR, not recorded

^1^Routine endometrial sampling from 2006 onwards; ^2^1EC on incident screening; ^3^Incidental finding of EC at prophylactic hysterectomy in a woman newly diagnosed with colorectal cancer by colonoscopy surveillance; ^4^4 AEH/EC found at RRS despite negative TVS; ^5^Colonoscopy done at the same time; ^6^One EC associated with OC detected by ultrasound; ^7^January 2003-December 2007 hysteroscopy and biopsy only performed on clinical indication; ^8^January 2008-June 2012; ^$^Mixed-Lynch syndrome, Lynch-like syndrome, strong family history of Lynch syndrome, families fulfilling Amsterdam II criteria; *Overlap in included patients

Key studies, summarised in Table 2, show that gynaecological surveillance can detect both symptomatic and asymptomatic gynaecological (pre)malignancies, however, the absolute numbers of atypical endometrial hyperplasia, endometrial and ovarian cancers identified were low.

In a retrospective cohort of patients with Lynch syndrome with a gPV in *MSH2*, few endometrial cancer cases were observed in the surveillance compared with non-surveillance groups [28], but the reasons for this difference were unclear and may reflect confounding factors unaccounted for in the analysis. Overall, they found no evidence that surveillance reduced gynaecological cancer incidence or improved cancer-specific outcomes, and ovarian cancer deaths occurred despite surveillance. In several studies with surgical comparator groups, endometrial cancers were detected at the time of RRS in women who had previously participated in surveillance, suggesting missed or interval cancers [32, 34, 35]. Other studies reported interval or missed cancers predominantly in symptomatic women in the surveillance group, although absolute rates were low. De Jong et al. found that the introduction of a gynaecological surveillance programme did not reduce endometrial cancer–related mortality [37]. In a large single-centre retrospective cohort, Duenas et al. reported lower endometrial and ovarian cancer specific mortality, as well as all-cause mortality, in those undergoing RRS compared to surveillance, although these estimates were constrained by small sample size and limited duration of follow up [34]. In a nationwide retrospective cohort of 1,046 patients with Lynch syndrome, Eikenboom et al. found that although gynaecological surveillance was associated with higher detection rates of hyperplasia and endometrial cancer at slightly earlier stages, it did not reduce adjuvant treatment requirements or improve survival; five-year overall survival was similar between surveillance and non-surveillance groups (97% (95% CI 90–100%) vs. 87% (95% CI 70–100%)) [36]. These findings should be interpreted with caution given the study’s retrospective design, lack of adjustment for key confounders, including younger age and a higher proportion of high-risk genotypes (*MLH1*, *MSH2*, *EPCAM*) in the surveillance group, and evolving surveillance practices over the study period.

It remains difficult to draw firm conclusions about the effectiveness of gynaecological surveillance because of substantial heterogeneity in study design, variation in surveillance modalities and programmes, failure to adequately control for confounding and the inherent selection bias of retrospective cohorts. Although some studies included surgical comparator groups, baseline differences between the surveillance and surgical cohorts were not sufficiently adjusted for, limiting the validity of these comparisons. Several studies also counted cancers diagnosed in symptomatic women, who may have presented and been diagnosed in routine care even without surveillance. Gynaecological surveillance protocols varied widely, particularly in the diagnostic modalities used, and some cohorts included individuals without proven Lynch syndrome, further limiting generalisability of findings to genetically confirmed Lynch syndrome populations.

Despite the lack of evidence for the clinical effectiveness of endometrial surveillance for detection of premalignant and early-stage endometrial cancer, patients with Lynch syndrome perceive surveillance to be reassuring and acceptable [38–40]. A prospective study looking at acceptability of endometrial surveillance in patients with Lynch syndrome found that most women invited for surveillance accepted the invitation, with only seven out of 37 declining, mostly because they were trying to conceive [39].

The optimal modality and interval for gynaecological surveillance remains undefined. Transvaginal ultrasound is generally preferred by patients, as it is less invasive and associated with significantly less discomfort than outpatient hysteroscopy or pipelle biopsy [39]. However, in premenopausal women, who make up the majority of those undergoing surveillance, transvaginal ultrasound is limited by lower specificity due to natural cyclical changes in endometrial thickness [41, 42]. Ultrasound alone performs poorly as a surveillance tool in this population and the addition of endometrial biopsy improves the detection of endometrial cancer and premalignancies [18, 23]. Most women report mild or intermediate pain with endometrial biopsy [38, 43], although postmenopausal women have higher pain scores [43], and no significant difference in pain scores have been observed in hysteroscopy and pipelle biopsy [39]. Data from general population indicate that blind endometrial sampling may miss 50–85% of intracavity pathology such as endometrial polyps [44, 45] and is associated with 11% failure rate and 31% risk of inadequate sampling [46], often attributed to cervical stenosis or nulliparity. In cases of failed sampling, there is a 7% risk of finding significant pathology [46], warranting further investigation with hysteroscopy and endometrial biopsy under general or (regional) anaesthesia, which may increase anxiety and expose women to more harms associated with invasive procedures. Hysteroscopy allows direct visualisation of the endometrial cavity to enable directed biopsy or removal of focal lesions and can be performed in an out-patient or in-patient setting under local, general, or regional anaesthesia. In patients with Lynch syndrome, studies using hysteroscopy combined with endometrial biopsy in an outpatient setting have reported sensitivities of 100% for the detection of endometrial cancer [27, 47]. Nonetheless, outpatient hysteroscopy is associated with pain, sub-optimal visualisation of the uterine cavity and procedural abandonment due to cervical stenosis necessitating completion under anaesthetic [48]. These limitations underscore the challenges of current surveillance modalities. While such strategies continue to be undertaken in clinical practice, there is a need for a non-invasive surveillance test that could effectively triages women to diagnostic procedures and reduces the burden of repeated invasive investigations. The combination of colonoscopy and endometrial sampling under conscious sedation has been explored and shown to result in significantly less pain than outpatient endometrial sampling, but has logistical challenges [31, 49].

A survey of women from Lynch syndrome families attending gynaecological surveillance, including mutation carriers, women meeting Amsterdam II criteria, obligate carriers and first-degree relatives, reported that surveillance was not associated with increased psychological morbidity such as anxiety or depression [50]. However, the study included a small sample (*N* = 26), and only 58% of participants completed follow-up questionnaires. Notably, the potential harms and psychological burden associated with repeated gynaecological surveillance procedures have not been systematically evaluated. The cost-effectiveness of gynaecological surveillance in Lynch syndrome remains uncertain. Previous economic evaluations have primarily compared surveillance with RRS, rather than assessing its value in women who are unable or unwilling to undergo RRS. Yang et al. reported RRS to be the most cost-effective strategy, with an estimated cost of US$23,422 per patient for 25.71 quality-adjusted life years (QALYs), compared with US$68,392 for 25.17 QALYs with annual gynaecological surveillance [51]. A combined strategy of annual surveillance from age 30 followed by prophylactic surgery at age 40 years offered the greatest health benefit by delaying the consequences of premature menopause, but was not cost-effective due to the cumulative cost of 10 years of surveillance [52]. These studies are limited by the use of effectiveness estimates largely derived from postmenopausal women without Lynch syndrome, which may introduce bias and by their conduct within the US healthcare system, which may limit generalisability to other settings. More recently, Snowsill et al. used a disease simulation model incorporating gene-specific cancer risks, quality-of-life effects and incomplete uptake of surgery. In contrast to earlier models, their analysis suggested that strategies offering surveillance, either alone or alongside the option of later RRS, may be cost-effective for patients with gPVs in *MLH1*, *MSH2* or *MSH6*, largely reflecting assumptions that not all women undergo immediate surgery and that premature surgical menopause may reduce quality-adjusted life expectancy. However, RRS was still predicted to provide substantial reductions in cancer risk and mortality, and for patients with gPVs in PMS2, neither surveillance nor RRS was found to be cost-effective [53].

## Risk-reducing surgery

Risk-reducing hysterectomy with bilateral salpingo-oophorectomy is the most effective strategy for preventing gynaecological cancer in patients with Lynch syndrome. However, premenopausal risk-reducing hysterectomy with bilateral salpingo-oophorectomy is associated with premature menopause, which is detrimental to long-term health and quality of life. Women commonly experience vasomotor symptoms, sleep disturbance, mood changes, reduced libido, sexual dysfunction and loss of bone mineral density [54, 55]. Evidence from the general population also links premature menopause to increased risk of cardiac disease, stroke and neurocognitive impairment and reduced life expectancy [56]. However, much of the evidence describing the long-term consequences of risk-reducing oophorectomy derives from populations of patients with *BRCA1*/2 gPVs, many of whom have a personal history of breast cancer and may have limited access to hormone replacement therapy (HRT), which may affect the generalisability of these findings to patients with Lynch syndrome. HRT is therefore recommended until the natural age of menopause to alleviate menopausal symptoms and mitigate the long-term sequelae of surgical menopause in women without contraindications.

Decisions about RRS are strongly influenced by age, desire for fertility, menopausal status, personal history of cancer and a strong family history of malignancy [57]. An international survey of 31 centres across 18 countries found that, although most centres offered RRS, typically from around 40 years of age, there was marked variation in the timing of surgery, particularly for patients with a gPV in *PMS2* and in the provision of HRT after surgery [58]. Only 67% of patients with a gPV in *PMS2* were offered RRS, and only 71% of centres reported routinely offering oestrogen-only HRT to premenopausal women undergoing hysterectomy with bilateral salpingo-oophorectomy, although this may reflect variation in patient eligibility for HRT. While some of this variation likely reflects the lower cancer risk associated with *PMS2* and the greater flexibility in guideline recommendations for this genotype, these findings nevertheless illustrate differences in clinical practice regarding surgical risk reduction strategies and HRT provision for patients with Lynch syndrome.

Data from the Prospective Lynch Syndrome Database (PLSD) indicate that performing RRS before 40 years of age offers only limited additional reduction in endometrial cancer incidence and mortality for patients with a gPV in *MLH1*, *MSH2* and *MSH6*, and no clear benefit for patients with a gPV in *PMS2* [59]. For example, hysterectomy at age 40 years was estimated to prevent endometrial cancer before age 50 in approximately 13%, 16% and 11% of patients with gPVs in *MLH1*, *MSH2* and *MSH6* respectively, compared with 15%, 18% and 13% if performed at age 25 years, with no benefit observed for *PMS2*. Accordingly, RRS is recommended from 40 years of age for patients with gPVs in *MLH1*, *MSH2* and *MSH6*, and deferred until approximately 50 years of age for those with gPVs in *PMS2*.

Decisions about RRS should be made within a multidisciplinary setting. In particular, when women with Lynch syndrome require colorectal surgery, joint consultation with a gynaecologist allows concomitant hysterectomy and bilateral salpingo-oophorectomy to be considered, potentially avoiding multiple abdominal procedures and reducing cumulative surgical risk.

Hysterectomy with ovarian conservation is generally not recommended in Lynch syndrome because of the lifetime risk of ovarian cancer. However Lynch syndrome-associated ovarian cancer often presents at an earlier stage with better prognosis than the general population, likely reflecting the predominance of endometrioid and clear cell subtypes rather than high grade serous cancers [48]. Current evidence indicates that ovarian cancer risks vary by gene. Data from the PLSD suggest that premenopausal prophylactic oophorectomy does not confer a measurable mortality benefit in patients with gPVs in *MSH6* and *PMS2*, supporting consideration of ovarian conservation with delayed oophorectomy in selected patients [59].

Although data to guide delayed oophorectomy are limited, staged approaches have been proposed in women in which bilateral salpingectomy is performed at the time of hysterectomy with delayed oophorectomy to avoid premature menopause [60]. This concept is based on evidence from the general population reflecting that many high grade serous ovarian cancers arise from the fallopian tube. Observational studies have reported that opportunistic salpingectomy in the general population is associated with a 42–64% reduction in ovarian cancer risk [61, 62]. However, the extent to which these data apply to Lynch syndrome remain uncertain, particularly given the predominance of non-serous ovarian histotypes which may arise through different biological pathways [63]. Consequently, the potential protective effect of salpingectomy in Lynch syndrome remains unclear and requires further study.

Further studies have explored the impact of RRS in patients with Lynch syndrome. Moldovan et al. reported that, although most women did not experience significant psychological distress or decisional regret following RRS, many felt inadequately prepared for the consequences of premature menopause and described inconsistent advice regarding the initiation and duration of HRT [64]. Garg et al. identified pre-operative counselling about HRT as a key modifiable factor associated with uptake [65]. For patients with Lynch syndrome considering RRS, these findings underscore the importance of comprehensive pre-operative counselling led by clinicians with expertise in Lynch syndrome, proactive management of menopausal symptoms, and clear, consistent guidance on HRT, delivered within multidisciplinary care pathways that also consider the option of concomitant RRS when other abdominal procedures are planned.

## Minimally invasive biomarkers

Current surveillance methods for detecting endometrial cancer in patients with Lynch syndrome with transvaginal ultrasound and endometrial biopsy are invasive, uncomfortable or even painful [66]. Gynaecological surveillance to detect ovarian cancer at an early stage is not proven to be effective at reducing deaths form the disease [67]. This raises the need for minimally or non-invasive approaches for gynaecological cancer surveillance [68].

Cervicovaginal specimen biomarkers have emerged as a promising field for the detection of endometrial and ovarian cancer in the general population. Among these, DNA methylation analysis of cervical swabs seems to be the most promising approach to detect gynaecological cancers, although further prospective validation is required [69].

A systematic review of 45 studies including 6,599 patients with endometrial cancer found abnormal cervical cytology in 45% (95% CI 40%-50%) of women prior to diagnosis and surgery for endometrial cancer [70]. Endometrioid tumours were less frequently picked up by cytology than non-endometrioid tumours (44% (95% CI, 34–53%) versus 77% (95% CI, 66–87%)), limiting the applicability of cervical cytology for gynaecological surveillance in Lynch syndrome.

Using cervical smear specimens, Kinde et al. demonstrated that mutations identified through whole-gene panel analysis in cervical smears had a sensitivity of 100% for endometrial cancer and 41% for ovarian cancer detection [71]. In a large case-control study, Wang et al. introduced PapSEEK, a cervical screening test that combines assays for identifying mutations in 18 genes as well as an assay for aneuploidy [72]. The test demonstrated a sensitivity of 81% in 382 patients with endometrial cancer, and a sensitivity of 33% in 245 patients with ovarian cancer. Among 714 healthy women, only 1.4% yielded a positive PapSEEK result, indicating in high specificity of the test.

Barrett et al. investigated the use of CpG site methylation in cervical smears to identify patients with endometrial or ovarian cancer [73]. Their assay demonstrated an area under the curve (AUC) of 0.81 for endometrial cancer and of 0.76 for ovarian cancer. In a separate study, the methylation array identified endometrial cancer with a sensitivity of 86% and specificity of 90% in an external validation cohort [74]. In a prospective validation cohort of 150 women without Lynch syndrome, the test achieved sensitivity and specificity of 52% and 98%, respectively, supporting its potential to identify women with, or at increased risk in the future of, endometrial cancer.

Methylation of *GYPC* and *ZSCAN12* in cervical smears, and (self-collected) vaginal swabs was also evaluated for the detection of endometrial cancer [75]. In multiple cohorts of symptomatic patients, it detected endometrial cancer with sensitivities exceeding 90%. This is the only study to include a cohort of patients with Lynch syndrome; among 25 patients with Lynch syndrome, the sensitivity to detect endometrial cancer was 33%, while specificity reached 100%.

Vaginal fluid and voided urine samples offer the opportunity for non-invasive detection of endometrial cancer through self-collection at home and postal return to the laboratory for testing. In a proof of principle case control study, urogenital cytology had a sensitivity of 92% and specificity of 89% for endometrial cancer in symptomatic patients [76]. Infrared spectroscopy and attenuated total reflection technology distinguished endometrial cancer from benign gynaecological conditions in urine samples with a sensitivity of 98% and specificity of 97% [77].

In recent years, there has been increasing interest in the composition of the gut microbiome because of its potential role in the pathogenesis of sporadic colorectal cancer [78, 79]. Moreover, studies have identified differences in the composition of the gut microbiome and specific bacterial operational taxonomic units overrepresenting between patients with Lynch syndrome (with or without a history of colorectal cancer) and non-Lynch syndrome patients [80, 81]. These finding raise the question of whether similar differences exist in the composition of the vaginal microbiome between patients with and without Lynch syndrome.

To date, no data are available regarding the composition of the vaginal microbiome patients with Lynch syndrome [69]. However, in healthy women without endometrial cancer, the vaginal microbiome is typically rich in *Lactobacillus* species, exhibits high microbial diversity, and maintains a low pH. In contrast, women with endometrial cancer tend to have a *Lactobacillus*-poor, less diverse microbiome with an elevated pH [82–84]. Similarly, a *Lactobacillus*-poor vaginal microbiome has also been observed in women with ovarian cancer [85, 86]. These findings suggest a potential role for the vaginal microbiome in the pathogenesis of gynaecological cancers in patients with Lynch syndrome and could provide biomarkers for their early detection, but further research is warranted.

In conclusion, DNA methylation analysis of cervicovaginal swabs appears to be a promising minimally invasive technique for the detection of gynaecological cancers. However, no large cohort studies have yet been conducted in women with Lynch syndrome. Therefore, prospective studies specifically involving women with Lynch syndrome are warranted.

## Chemoprevention

### Hormonal agents

Sporadic, oestrogen-dependent endometrial cancer is known to be associated with obesity [87]. Approximately half of all endometrial cancer cases in postmenopausal women are related to obesity [88]. In women living with obesity, increased conversion of androstenedione to estrone and aromatization of androgens to estradiol in adipose tissue lead to elevated levels of endogenous oestrogen [89]. In the absence of adequate progesterone counterbalance, these endogenous oestrogens increase proliferation of the endometrium, which may lead to endometrial hyperplasia, atypia and/or finally endometrial cancer [90].

In the general population, the use of oral contraceptives reduces the risk of endometrial cancer and, to a lesser extent, ovarian cancer [91, 92]. The use of a levonorgestrel-releasing intrauterine system (LNG-IUS) is also associated with a decreased risk of developing endometrial cancer and is used as a treatment of endometrial hyperplasia [93, 94]. In the prospective Norwegian Women and Cancer Study (NOWAC), the association between LNG-IUS use and risk reduction of endometrial and ovarian cancer was evaluated in a cohort of almost 105,000 women. The relative risks of developing endometrial and ovarian cancer among LNG-IUS users were 0.22 (95% CI: 0.13–0.40), and 0.53 (95% CI: 0.32–0.88), respectively [95].

There are no data on the use of intrauterine contraceptives in patients with Lynch syndrome to lower the risk of endometrial or ovarian cancer. The POET trial, which investigated the efficacy of an LNG-IUS for the prevention of endometrial atypia or cancer in patients with Lynch syndrome, was stopped in 2015 due to a lack of participants [96]. Fertility-sparing management of early-stage endometrial cancer with oral progestins, with or without an LNG-IUS, has demonstrated reduced efficacy in mismatch repair-deficient tumours [97]. This observation may be attributable to different pathogenesis of mismatch repair deficient and oestrogen-dependent endometrial cancer. Evidence regarding risk reduction of endometrial or ovarian cancer through contraceptive use in Lynch syndrome is limited. Some studies have demonstrated a reduction of endometrial proliferation or endometrial cancer among patients with Lynch syndrome using oral contraceptives or depo-medroxyprogesterone acetate injection [98, 99]. Dashti et al. demonstrated a reduced risk of developing endometrial cancer in patients with Lynch syndrome, with a hazard ratio of 0.39 (95% CI: 0.23–0.64) among women who had ever used hormonal contraceptives compared with never-users. However, this was a retrospective cohort of just 1,128 women. This suggests that oral contraceptives or depot medroxyprogesterone acetate may represent feasible chemoprevention strategies for endometrial cancer in patients with Lynch syndrome, particularly those wishing to preserve fertility for future childbearing.

### Aspirin

The use of aspirin may reduce the risk of developing Lynch-related cancers, especially colorectal cancer [100, 101]. The exact mechanism by which aspirin exerts its preventive effect on colorectal carcinogenesis remains not fully understood. Currently it is suggested that aspirin inhibits cyclo-oxygenase (COX) enzymes, leading to a reduction in prostaglandin E₂ levels [101]. This decrease suppresses angiogenesis, cellular proliferation, and tumour cell survival. In addition, aspirin may induce upregulation of CD80 expression on antigen-presenting cells, thereby enhancing their capacity to present tumour-associated antigens to cytotoxic T cells. This immunomodulatory effect could facilitate immune recognition and elimination of transformed cells, contributing to aspirin’s chemopreventive activity [102].

The double-blind, randomised CAPP2 study demonstrated a reduction in colorectal cancer incidence among patients with Lynch syndrome with the use of 600 mg of aspirin daily for two years, without major side effects [103]. There was no significant reduction in the incidence of endometrial or ovarian cancers in CAPP2, most likely due to insufficient numbers of people at risk for these cancers included in the study. The optimal dose of aspirin will be evaluated in the ongoing CAPP3 study. The use of aspirin is therefore recommended for the prevention of Lynch syndrome related cancers overall and can be used alongside other approaches for gynaecological cancer prevention [100, 104].

## Vaccination

Patients with Lynch syndrome may benefit from immune-supportive strategies such as vaccination to prevent cancer [105]. In Lynch syndrome, mismatch repair deficiency results in the inability to correct base-pairing errors that occur during DNA replication, leading to the accumulation of mutations within microsatellite regions. This phenomenon is known as microsatellite instability (MSI) [106]. MSI also frequently induces insertion and deletion mutations in coding microsatellites, generating frameshift peptides (FSPs). These FSPs can give rise to novel protein sequences, or neoantigens (neoAgs), which are absent in normal tissues and thus recognized as foreign by the immune system [107]. NeoAgs derived from FSPs are highly immunogenic and capable of eliciting robust T-cell-mediated immune responses [108]. Vaccination with these FSP-derived neoantigens has the potential to prime the immune system to recognize and eliminate cells expressing these tumour-specific antigens, thereby preventing cancer initiation and progression [109].

A phase I/IIa clinical trial published in 2020 demonstrated that vaccination with FSP neoAgs in patients with MSI colorectal cancer is systemically well tolerated and consistently induces both humoral and cellular immune responses [109]. In a mouse model, vaccination with a combination of four FSPs neoAgs significantly increased FSP-specific adaptive immunity, reduced intestinal tumour burden and prolonged overall survival. This pre-clinical study supports a clinical strategy of FSP neoAg vaccination for cancer prevention in patients with Lynch syndrome [110]. In the ongoing trial ‘*A Phase Ib/II Clinical Trial of Nous-209 for Recurrent Neoantigen Immunogenicity and Cancer Immune Interception in Lynch Syndrome’*, the safety and efficacy of a neoAg vaccine are being evaluated [111]. The study aims to assess whether this vaccination can influence the development of colorectal polyps or tumours, with results expected by the end of 2026.

Another phase I/II open-label study is evaluating the safety and immunogenicity of vaccination with frameshift-derived neoantigen (neoAg)–loaded dendritic cells [112]. Patients with Lynch syndrome with colorectal cancer, patients with MSI-positive colorectal cancer but unknown or no germline mutation, and patients with Lynch syndrome without cancer will be vaccinated. The primary objective is to evaluate toxicity, while secondary aims include determining whether the vaccine can induce or enhance immune responses against selected frameshift-derived neoAgs and evaluating associated pathological and clinical effects. Study results are expected soon.

Another ongoing vaccine trial is investigating whether the combination of a trivalent adenovirus-5 (Tri-Ad5) vaccine and the IL-15 super agonist N-803 can reduce the incidence of colorectal cancer in patients with Lynch syndrome [113]. The Tri-Ad5 vaccine encodes tumour-associated antigens that are overexpressed in premalignant and malignant tissues. N-803 may increase immune responses to other vaccines. Giving Tri-Ad5 in combination with immune enhancing N-803 may lower the chance of developing colon and other cancers in participants with Lynch syndrome. The estimated completion date of this study is 2028.

Although vaccination studies for Lynch syndrome-related cancer prevention appear promising, current clinical trial data remain insufficient and are primarily focused on colorectal cancer.

## Lifestyle

Lifestyle has been identified as an important risk factor to various types of cancer in the general population [114]. Healthier lifestyle changes are associated with a reduced all-cause and cancer-specific mortality [115]. However, evidence regarding lifestyle factors on Lynch-related gynaecological cancers is limited. In three studies evaluating weight status or weight change in relation to endometrial cancer risk in patients with Lynch syndrome, no significant association was observed [116]. Similarly, a retrospective cohort study involving 136 patients with Lynch syndrome found that body mass index, smoking or alcohol intake did not influence endometrial cancer risk [117].

In a recent systematic review and meta-analysis, the risk of colorectal cancer was significantly higher in patients with Lynch syndrome living with obesity, compared with individuals with a healthy weight (adjusted hazard ratio 2.38, 95% CI: 1.52–3.72) [118]. Additionally, self-reported physical activity was associated with a reduced risk of colorectal cancer in two studies [119, 120]. No significant associations were observed for smoking or alcohol consumption [118].

Obesity, impaired physical functioning, and poor nutritional status are established risk factors for perioperative and postoperative complications in gynaecological cancer surgery [121]. Although no studies clearly showed that lifestyle modification reduces the risk of gynaecological cancer in patients with Lynch syndrome, adopting a healthy lifestyle is recommended. This includes following a balanced diet, engaging in regular physical activity, maintaining a healthy body weight, avoiding smoking, and limiting or abstaining from alcohol consumption [5, 104].

## Conclusion and future perspectives

Despite substantial advances understanding the carcinogenesis in Lynch syndrome, optimal strategies for gynaecological cancer prevention remain understudied. Emerging data highlight the need to move from a uniform approach to personalised model that balances individual cancer risk-reduction with quality of life.

Progress in the prevention of gynaecological cancer in Lynch syndrome relies on personalised multidisciplinary care. Key priorities include developing standardised, gene and age specific protocols for RRS and post-operative HRT regimes. Incorporating patient preferences throughout these pathways will help deliver more effective, less burdensome and individualised care.

Gynaecological prevention strategies must be firmly grounded in the experiences and preferences of patients with Lynch syndrome. Decision-making around gynaecological cancer prevention strategies is complex, requiring women to balance cancer risk, fertility wishes, implications of surgical menopause and the burden of repeated invasive diagnostics. There is a particular need for large-scale prospective work exploring effectiveness of attitudes to surveillance and RRS, including reasons for non-attendance and perceived harm of different surveillance approaches. These insights can inform decision aids, counselling frameworks and service models that support joint decision making for women at risk of gynaecological cancer in Lynch syndrome.

Robust evaluation of benefits, harms and cost-effectiveness of current prevention strategies such as surveillance, alongside the development of accurate minimal or non-invasive alternatives, and further work on preventative cancer vaccines, are essential to reduce morbidity and mortality among patients with Lynch syndrome.

## Data Availability

No datasets were generated or analysed during the current study.

## Declarations

### Corresponding author

Kevin JJ Kwinten, Department of Obstetrics and Gynecology, Radboud University Medical Center, Nijmegen, The Netherlands, kevin.kwinten@radboudumc.nl.
